# *Bordetella pertussis* infection activates the type I interferon signaling pathway to exacerbate respiratory tract inflammatory response

**DOI:** 10.3389/fimmu.2025.1521970

**Published:** 2025-03-07

**Authors:** Wenwen Jiang, Jiangli Liang, Lukui Cai, Jingyan Li, Qin Gu, Yan Ma, Mingbo Sun, Xin-an Jiao, Li Shi

**Affiliations:** ^1^ Jiangsu Key Laboratory of Zoonosis, Jiangsu Co-Innovation Centre for Prevention and Control of Important Animal Infectious Diseases and Zoonoses, Yangzhou University, Yangzhou, China; ^2^ Department of Laboratory Medicine, Affiliated Hospital, Yangzhou University, Yangzhou, China; ^3^ Laboratory of Vaccine Development, Institute of Medical Biology, Chinese Academy of Medical Science & Peking Union Medical College, Kunming, Yunnan, China; ^4^ Laboratory of Immunogenetics, Institute of Medical Biology, Chinese Academy of Medical Science & Peking Union Medical College, Kunming, Yunnan, China

**Keywords:** *Bordetella pertussis*, RNA sequencing, transcriptome analysis, type I IFNs, IFNAR1

## Abstract

The upper airway epithelium is the primary site of exposure to *Bordetella pertussis* and the initiator of host responses to this microbe. *B. pertussis* may cause serious respiratory infections resulting in major complications, as well as severe impairment of airway function. While pertussis treatment options are limited because the molecular responses in the upper respiratory tract in the early stages of infection have not been characterized. Here, we performed a genome-wide transcriptional analysis of nasal turbinates isolated from mice over 11 days after *B. pertussis* infection. Using RNA-seq, we characterized the differentially expressed genes and pathways associated with the changes in the nasal turbinates following infection. Statistical analysis revealed that infection is a dynamic process characterized by increased expression of a set of acute inflammatory responses at an early stage. After this initial inflammatory response, we observed increases in the levels of transcripts associated with the regulation of immune processes. In particular, we found that *B. pertussis* infection significantly increased the levels of type I interferons (IFNs) and related genes in the nasal turbinates at 2 h, 2 days, and 4 days postinfection. Therefore, we investigated the role of type I IFNs in *B. pertussis* infection in type I IFNs receptor-deficient (IFNAR1^−/−^) mice. There was no difference in bacterial clearance or adaptive immune responses between wild-type and IFNAR1^−/−^ mice. However, a lack of type I IFNs signaling ameliorated pulmonary immunopathology, reduced the production of inflammatory cytokines and limited the recruitment of neutrophils to the lung during *B. pertussis* infection. Thus, our findings suggest that inhibiting the effects of type I IFNs may contribute to dampening inflammation, which could be an approach for the treatment of *B. pertussis* infection and management of the associated disease symptoms.

## 1 Introduction

Whooping cough, or pertussis, is a highly contagious respiratory illness caused by *Bordetella pertussis* and remains a lethal threat for at-risk individuals, particularly unvaccinated infants (1). Pertussis is a preventable disease; however, it has experienced a resurgence during recent decades, despite high vaccine coverage (2, 3). In particular, following the COVID-19 pandemic, several countries have experienced pertussis outbreaks and dramatic increases in pertussis-related deaths, drawing significant clinical attention (2, 4, 5). A major challenge in the prevention and treatment of pertussis is our relatively poor understanding of the mechanisms of disease. In addition, the inefficient protection afforded by current acellular pertussis vaccines (aP), as well as a lack of effective treatments, especially as macrolide resistance has been reported, contribute to these challenges (6, 7). Research on the mechanisms of pertussis pathogenesis can help identify effective therapeutic targets, which in turn could benefit individuals with severe disease and save the lives of infected infants.

*B. pertussis* is known to be mainly an upper respiratory tract pathogen that can colonize the human upper respiratory tract by attaching to ciliated cells, but it can eventually infect the tracheobronchial tree and progress to lower respiratory tract infection, especially in severe cases (8–10). In the initial (catarrhal) phase of pertussis is indistinguishable from common upper respiratory infections. It includes nasal congestion, rhinorrhea, and sneezing, variably accompanied by low-grade fever, and catarrhal stage is most infectious (11). It is well-known that the nasal mucosa is often the initial site of respiratory pathogen infection, replication, and transmission. In the presence of pathogens, the nasal mucosa protects against infection and limits spread to the lower respiratory tract via a variety of mechanisms. The nose is an important component of the mucosal immune system that can function as an immunologic sensor to detect danger signals, including from pathogens (12). Recent studies have indicated that impaired nasal epithelial antiviral immunity may underlie and precede severe COVID-19 (13). In addition, Hua et al. (14) reported that the activation of the local nasal mucosal immune system can impact the lung immune environment. For example, the nose remotely primes lung immunity to protect the lungs from direct viral infections (14). Current studies on the pathogenesis of pertussis infection have focused on lung tissue; however, there are few data available describing the upper airway immune responses to *B. pertussis* infection.

To better understand the molecular responses in the upper respiratory tract in the *B. pertussis*, we profiled the nasal turbinate transcriptomes of mice infected with *B. pertussis* via RNA-seq. We found that *B. pertussis* infection altered the expression of hundreds of genes, in particular, increasing the expression of a set of early inflammatory and innate immune response genes. Surprisingly, the results showed that *B. pertussis* infection induces an intense type I IFNs response, which motivated us to further investigate the role of type I IFNs signaling in *B. pertussis* infection. Although usually considered to be most important in the response to viruses, type I IFNs are also induced by most, if not all, bacterial pathogens. Although multiple mechanisms have been described, bacterial induction of type I IFNs occurs upon stimulation of two main pathways: (1) Toll-like receptors (TLRs) recognition of bacterial molecules such as lipopolysaccharide (LPS), RNA, and unmethylated CpG DNA; (2) TLR-independent recognition of molecules, like Nod-like receptors (NLRs), retinoic acid-inducible gene I (RIG-I)-like receptors (RLRs), the recently described receptor cytosolic GAMP synthase (cGAS), and other cytosolic nucleic acid sensors (15, 16). These receptors transduce signals downstream through a few key molecules such as the IFN-regulatory factor (IRF) family of transcription factors, resulting in the production of type I IFNs. After production, type I IFNs activate a wide range of gene transcription through downstream classical JAK (Janus activated kinase)–STAT (signal transducer and activator of transcription) pathway of signaling and non-canonical pathway such as phosphoinositide 3-kinase (PI3K)–mammalian target of rapamycin (mTOR) pathway and multiple mitogen-activated protein kinase (MAPK) pathways (17).

The antiviral function of type I IFNs has been extensively documented, but the role of type I IFNs in the response to bacterial infection is complex and can be either deleterious or protective depending on the nature of the pathogen (16, 18). To better understand the functional consequences of type I IFN signaling during *B. pertussis* infection, we analyzed mice deficient in type I IFN receptors in the context of *B. pertussis* infection.

## 2 Materials and methods

### 2.1 Mice and ethics statements

Specific pathogen-free (SPF) 6- to 8-week-old male and female C57BL/6 mice were purchased from Beijing Charles River Laboratory (Beijing, China). Type I IFNs receptor-deficient (IFNAR1^−/−^) mice in a C56BL/6 background were originally obtained from The Jackson Laboratory. The animal work in this study was carried out in strict accordance with the Guide for the Care and Use of Laboratory Animals of the People’s Republic of China. All protocols were reviewed and approved by the Committee on Ethics of the Affiliated Hospital of Yangzhou University (2023-YKL02-G022).

### 2.2 Bacterial strains, media, and growth conditions

The *B. pertussis* strain *B.p-L1* used in this study was recently isolated from a patient in Yunnan Province, China (19, 20). The polymorphisms in the PT promoter (ptxP), PT subunit 1 (ptxA), and pertactin (prn) were assessed by DNA sequencing. The genotype of *B.p-L1* was ptxP3/ptxA1/prn2, consistent with the current epidemic strains. For *B. pertussis* challenge experiments, *B. pertussis* strain *B.p-L1* was grown on Bordet–Gengou (B-G) and Regan–Lowe plates prepared as described previously (19). Briefly, after 2 days of growth at 37°C, the bacteria were scraped off B-G blood agar plates, resuspended in phosphate-buffered saline (PBS), diluted to a concentration of 10^11^ CFU/mL via a turbidimetric method, and used for aerosol challenge. For the culture of bacteria from tissues, Regan–Lowe plates (Oxord) supplemented with 10% defibrinated sheep blood and 40 μg/mL cephalexin (Oxord) were used.

### 2.3 Mouse experiments

To investigate the transcriptional dynamics of murine nasal turbinates infected with *B. pertussis*, 6–8-week-old C57BL/6 mice (equal numbers of males and females) were infected with *B. pertussis* (10^11^ CFU/mL) via the aerosol method as described previously (19). Animals were selected for euthanization on day 1 (prechallenge, pre) and at 2 h and 2, 4, 7, and 11 days postinfection (hpi or dpi) for tissue collection.

To investigate the role of type I IFNs in *B. pertussis* infection, 6–8-week-old C57BL/6 mice (wild type, WT) and type I IFNs receptor-deficient (IFNAR1^−/−^) mice were also infected with *B. pertussis* (10^11^ CFU/mL) via the aerosol method as described previously (19). Animals were euthanized on day 1 (pre), 2 hpi and 2, 4, 7, 14, 21, and 28 dpi for tissue collection.

### 2.4 RNA sequencing and analysis

Nasal turbinates were collected on day 1 (pre), 2 hpi and 2, 4, 7, and 11 dpi and homogenized with TRIzol reagent (Invitrogen). Total RNA was extracted with chloroform/isopropanol, followed by purification via the RNeasy Mini Kit (Qiagen). Library construction and sequencing were performed as described previously (19, 21). Briefly, the mRNA was isolated and purified from total RNA via oligo(dT)-attached magnetic beads. The purified mRNA was subsequently fragmented into small pieces for cDNA synthesis. The quality of the cDNA library was assessed via an Agilent Technologies 2100 Bioanalyzer and qPCR. Paired-end sequencing with a read length of 100 bases was performed on the BGIseq500 platform. The raw data were filtered with SOAPnuke software (22). The clean data were mapped to the Mus-musculus_GRCm38.p6 reference genome with hierarchical indexing for spliced alignment of transcripts (HISAT) software (23). Fragments per kilobase of transcript per million mapped reads (FPKM) of each gene was calculated based on the length of the gene and RSEM (v1.2.12) were used to quantitative analysis (24). Before differentially expressed (DE) genes analysis, we conducted a Principal Component Analysis (PCA) analysis on raw counts using the factoextra package (v 1.0.7) (https://CRAN.R-project.org/package=factoextra) and the FactoMineR package (v 2.11) to verify the batch effect among the samples (**Supplementary Figure S1**, **Supplementary Table S2**). Differentially expressed (DE) genes were acquired using the Limma (v 3.48.3) package (p.adj < 0.05, |log2FC| > 1) (25). Venn diagram was drawn with Jvenn (http://jvenn.toulouse.inra.fr/app/example.html). Heatmaps were drawn with the heatmap (v1.0.12) R package. The enriched Gene Ontology (GO) and Kyoto Encyclopedia of Genes and Genomes (KEGG) pathways of the DE genes were analyzed via ClusterProfiler (4.0.5) in RStudio (26). And the bubbleplots were made and analyzed through R packages (27). To identify patterns of gene expression changes over the course of infection, the data were analyzed with the Short Time-series Express Miner (STEM) tool developed by Jason Ernst and Ziv Bar-Joseph (28).

### 2.5 Enzyme-linked immune sorbent assay

At four weeks after infection, *B. pertussis*-specific serum IgG titers were measured via ELISA as previously described (19). The components of pertussis antigens Pertussis toxoid (PT), filamentous hemagglutinin (FHA) and Pertactin (PRN) were purified by column chromatography as described previously (29). Specifically, PT, FHA, or PRN at 3 μg/mL were coated on microplates (96-well) overnight at 4°C. The plates were washed with wash buffer (0.05% (v/v) polysorbate 20 in PBS) once and then blocked with 3% (w/v) bovine serum albumin (BSA, Abcam) in wash buffer for 2 h at 37°C. The plates were subsequently washed and incubated with serially diluted mouse sera for 1 h at 37°C. After washing, the plates were incubated with horseradish peroxidase (HRP)-labeled sheep anti-mouse IgG (Jackson ImmunoResearch, USA) antibody for 1 h at 37°C. All the ELISA plates were developed with tetramethylbenzidine (TMB; Solarbio, CHN) to generate a colorimetric reaction, and the reaction was terminated with 2 mmol/L sulfuric acid. The absorbance of the plates at 450 nm was read. Endpoint titers were determined as the dilution that exhibited an optical density exceeding ≥ 2.1 times the background level (secondary antibody alone).

### 2.6 ELISA for cytokines and albumin

Cytokine levels in the bronchoalveolar lavage fluid (BALF) and the supernatant of cultured cells were measured with an ELISA kit according to the manufacturer’s recommendation. BALF was collected by flushing with 1 mL of PBS back and forth three times via a catheter and syringe as previously described (19). BALF samples were centrifuged at 800 × g for 10 min to obtain a cell pellet (for flow cytometry analysis) and supernatant (for cytokine analysis). The levels of TNF-α (Cat# VAL609, Novus Biologicals), IL-1β (Cat# SMLB00C, R&D Systems), IL-6 (Cat# VAL604, Novus Biologicals), and IFN-β (Cat# VAL612, Novus Biologicals) in the BALF were quantified. And the BALF albumin levels were measured by a mouse-specific albumin ELISA kit (ab207620, Abcam) according to the manufacturer’s instructions. To detect the cytokine levels in the supernatants of cultured splenic or pulmonary lymphocytes, the cells were cultured at a concentration of 2 × 10^6^/mL and stimulated with the antigens PT (2 μg/mL), FHA (2 μg/mL), and PRN (2 μg/mL). The supernatants were removed after 3 days and stored at -80°C before testing. IFN-γ (Cat# VAL607, Novus Biologicals) levels were measured for Th1 responses; IL-17A (Cat# VAL610, Novus Biologicals) levels were tested for Th17 responses; and IL-4 (Cat# M4000B, R&D Systems) levels were detected for Th2 responses.

### 2.7 Flow cytometry analysis

Single-cell suspensions were obtained from the BALF as described above. The BALF was centrifuged at 800 × g for 10 min to obtain a cell pellet, which was then washed twice with PBS. The cells were incubated with an anti-CD16/CD32 Fc block. Then, the cells were incubated with 7-AAD (BD Bioscience), followed by surface staining with fluorochrome-conjugated anti-mouse antibodies for the following markers. The neutrophils were identified as CD45+LY6G+ cells. All samples were acquired on a flow cytometer (Beckman), and the data were analyzed with FlowJo software (TreeStar).

### 2.8 Quantitative real-time PCR

Total RNA was extracted from nasal turbinates using TRIzol reagent (Invitrogen, USA) according to the manufacturer’s instructions. One microgram of RNA was used as the template to synthesize first-strand cDNA via a PrimeScriptTM RT kit (Accurate Biotechnology, CHN). cDNA was subsequently used as the template for real-time PCR using a SYBR Green Premix Pro Taq HS qPCR Kit (Accurate Biotechnology, CHN). The total reaction system was 20 μL, and the gene amplification process was performed with an Applied Biosystems 7500 Fast Real-Time PCR System (Life Technologies). The RNA expression of target genes was calculated via the 2^-ΔΔCt^ method on the basis of the housekeeping gene GAPDH. Every sample was analyzed three times in parallel. The primers used for the target genes are listed in **Supplementary Table S1**.

### 2.9 Histopathological analysis

For histopathological analysis, lung tissues from necropsied mice were fixed in 10% neutral buffered formalin, embedded in paraffin, and sectioned at 3–5 μm. Then, the sections were stained with hematoxylin and eosin (H&E) after dehydration. The pathological sections were observed and photographed under a microscope (Leica, Germany).

### 2.10 Statistical analysis

The data were graphed and analyzed via GraphPad Prism version 8.0 (GraphPad Software, Inc.). The results are presented as the means ± SEMs or GMTs and corresponding 95% confidence intervals (CIs). Unpaired Student’s t tests were applied to assess statistical significance. Differences were considered significant at P ≤ 0.05.

## 3 Results

### 3.1 Differentially expressed genes analysis highlights the dramatic initial response of the murine nasal turbinates to *B. pertussis* infection

**Figure 1:**
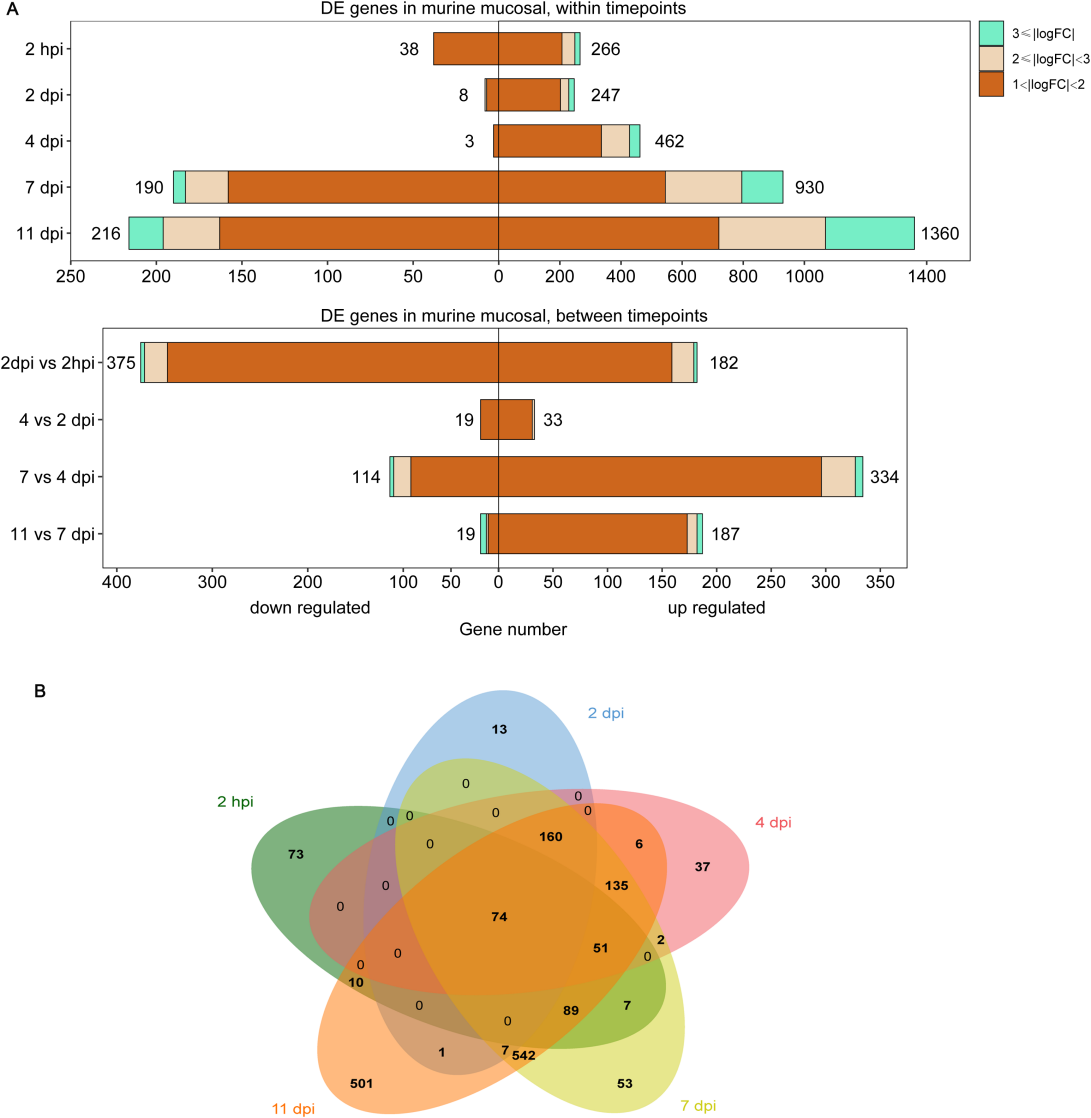
Differentially expressed (DE) genes in the murine nasal turbinates after *B. pertussis* infection. The nasal turbinates of mice infected with *B. pertussis* were sampled at day 1 (prechallenge, pre) and at 2 h and 2, 4, 7, and 11 days postinfection (hpi or dpi) for RNA-seq. **(A)** Pairwise comparisons were performed to identify differentially expressed (DE) genes between preinfection samples and samples collected at each timepoint (top) and between timepoints (bottom). **(B)** Venn diagram analysis was used to compare the DE gene lists for preinfected and infected mouse samples at each timepoint. The box length indicates the number of DE genes either downregulated (left) or upregulated (right) at an adjusted P value of < 0.05, with the total number of down- and upregulated genes shown. The color hue and shade indicate the proportion of genes with > 3-fold differential expression (cyan), between 2- and 3-fold differential expression (pink), or between 1- and 2-fold differential expression (orange), respectively.

Differential expression analysis was performed for a detailed analysis of the mouse nasal turbinates to *B. pertussis* infection at 2 h and 2, 4, 7 and 11 days after infection (hpi and dpi). Genes exhibiting significant changes in expression were identified via pairwise comparisons between each timepoint and the preinfection timepoint (2 hpi vs. pre, 2 dpi vs. pre, 4 dpi vs. pre, 7 dpi vs. pre, and 11 vs. pre). The total numbers of DE genes in these comparisons were 304 (266 upregulated, 38 downregulated), 255 (247 upregulated, 8 downregulated), 465 (462 upregulated, 3 downregulated), 1120 (920 upregulated, 190 downregulated), and 1576 (1360 upregulated, 216 downregulated) (**Figure 1A**, **Supplementary Table S2**). A direct comparison of the DE genes at each timepoint revealed that the nasal turbinate response to infection at 11 dpi was vastly different from the response at the early phase (**Figure 1B**). Among the 1576 DE genes at 11 dpi, 37% (501 genes) were unique to that of other detection timepoints. In contrast, 24% of the genes at 2 hpi (73 genes), 5% of the genes at 2 dpi (13 genes), 8% of the genes at 4 dpi (37 genes), and 5% of the genes at 7 dpi (53 genes) were differentially expressed only at that specific timepoint. To our interest, 74 genes (e.g., *Ccl5*, *Ccl2*, *Isg15*, *Ifitm3*, *Cd40*, *Cd53*, and et al.) were consistently up- or downregulated at all 5 timepoints (**Figure 1B**, **Supplementary Figure S2**).

To detect differences in gene expression over time, we conducted differential expression analysis across timepoints. Comparisons of successive timepoints revealed that the largest large number of genes exhibited changes in expression during the 2 hpi to 2 dpi transition (557 DE genes, 182 genes upregulated, 375 genes downregulated), followed by the 4 dpi to 7 dpi transition (448 DE genes, 114 genes upregulated, 334 genes downregulated), and relatively small changes between 2 and 4 dpi (52 genes, 33 genes upregulated, 19 genes downregulated) and between 7 and 11 dpi (206 genes, 19 genes upregulated, 187 genes downregulated) (**Figure 1A**, **Supplementary Table S2**). These findings suggest a strong initial response of the murine nasal turbinates to *B. pertussis* infection.

### 3.2 Functional enrichment analysis of DE genes following *B. pertussis* infection in murine nasal turbinates reveals robust inflammatory and immune responses

**Figure 2:**
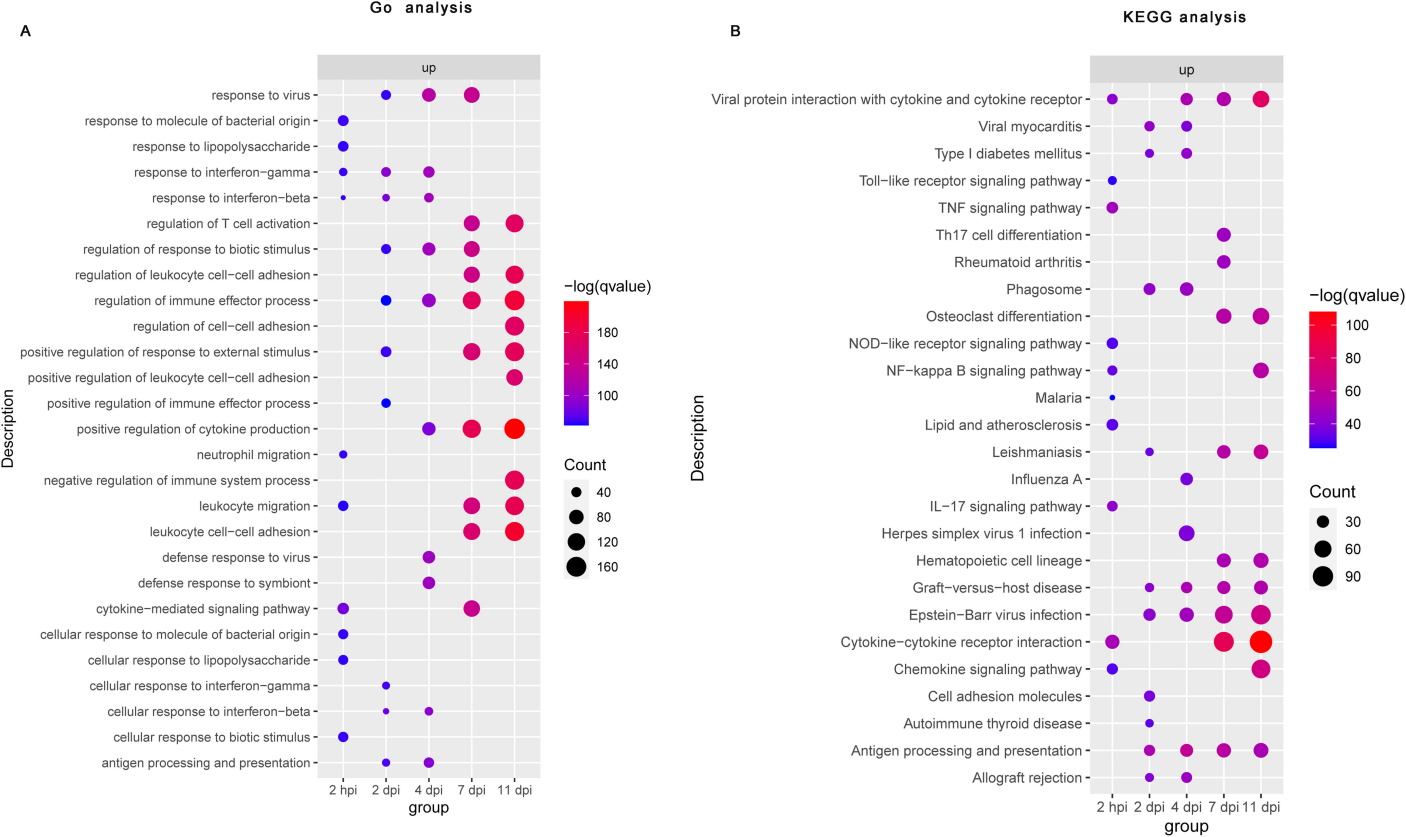
Functional enrichment analysis of differential gene expression following *B. pertussis* infection in murine nasal turbinates. Bubble plot summarizing the functional enrichment of DE genes upregulated at 2 hpi and at 2, 4, 7, and 11 dpi. The color intensity of each bubble represents the negative log of the FDR-adjusted p value [–log(q value)], and the relative size of each bubble represents the number of DE genes annotated with the specified Gene Ontology (GO) terms **(A)** and KEGG pathways **(B)**. The top 10 most significantly enriched GO terms and KEGG pathways related to the DE genes for each time point are shown.

To obtain an overview of the processes influenced by *B. pertussis* infection, we conducted GO and KEGG functional enrichment analyses of DE genes associated with the nasal turbinates at five timepoints. The top 10 results from each analysis were selected for visualization. GO enrichment analysis revealed that *B. pertussis* infection-upregulated genes detected at 2 hpi were enriched predominantly to GO terms related to inflammation and innate immunity, notably “response to lipopolysaccharide (LPS)”, “response to interferon-gamma”, “response to interferon-beta”, “neutrophil migration”, “leukocyte migration”, and “cytokine-mediated signaling pathways” (**Figure 2A**, **Supplementary Table S3**). However, at 2 and 4 dpi, the transcripts were enriched mainly in the GO terms related to immune regulation, including “regulation of immune effector process” and “antigen processing and presentation” (**Figure 2A**, **Supplementary Table S3**). Specifically, response to “interferon-gamma” and “interferon-beta” were enriched during the early stages of infection (2 hpi, 2 dpi, and 4 dpi). The DE genes upregulated at 7 dpi and 11 dpi were enriched predominantly to GO terms related to adaptive immunity, such as “regulation of T-cell activation” and the “regulation of leukocyte cell–cell adhesion” (**Figure 2A**, **Supplementary Table S3**). In contrast to the upregulated DE genes, a small number of downregulated genes were enriched in the early stages of infection (2 hpi and 4 dpi), and no pathways were enriched by GO enrichment analysis at 2 dpi (**Supplementary Figure S3A**, **Supplementary Table S3**). The GO terms enriched among genes downregulated in the later stages of infection were “fatty acid metabolic process”, “cellular response to xenobiotic stimulus”, “carboxylic acid biosynthetic process”, and so on (**Supplementary Figure S3A**, **Supplementary Table S3**).

Consistent with the GO enrichment analysis, the KEGG enrichment analysis revealed that upregulated DE genes detected at 2 hpi were enriched predominantly to KEGG pathway related to innate immunity and inflammation, like the “Toll-like receptor signaling pathway”, “NOD-like receptor signaling pathway”, “TNF signaling pathway”, “NF−kappa B signaling pathway”, “cytokine−cytokine receptor interactions”, and “chemokine signaling pathway” (**Figure 2B**, **Supplementary Table S4**). Interestingly, among the downregulated DE genes at 2 hpi, we did not observe any enrichment of the KEGG pathway (**Supplementary Figure S3B**, **Supplementary Table S4**). At 2 dpi, we observed strong positive enrichment of terms related to the “phagosome”, “cell adhesion molecules”, “antigen processing and presentation”, and so on (**Figure 2B**, **Supplementary Table S4**). Similarly, “phagosome” and “antigen processing and presentation” were also upregulated at 4 dpi (**Figure 2B**, **Supplementary Table S4**). However, a small number of downregulated genes were enriched at 2 dpi and 4 dpi (**Supplementary Figure S3B**, **Supplementary Table S4**). At 7 and 11 dpi, significantly upregulated DE genes were mainly enriched in adaptive immunity related pathways such as “Th17 cell differentiation”, “cytokine−cytokine receptor interaction”, “chemokine signaling pathway”, and “antigen processing and presentation signaling pathways”, whereas downregulated DE genes were enriched mainly in “steroid hormone biosynthesis”, “retinol metabolism”, “protein digestion and absorption”, and so on” (**Figure 2B**, **Supplementary Figure S3B**, **Supplementary Table S4**). These functional enrichment analyses suggest that nasal turbinates transcriptional response to *B. pertussis* infection revealed an acute upregulation of genes related to inflammation and innate immune activation and a later adaptive immune response.

### 3.3 Short time-series expression miner analysis of DE genes reveals the regulatory changes in the nasal turbinates after *B. pertussis* infection

**Figure 3:**
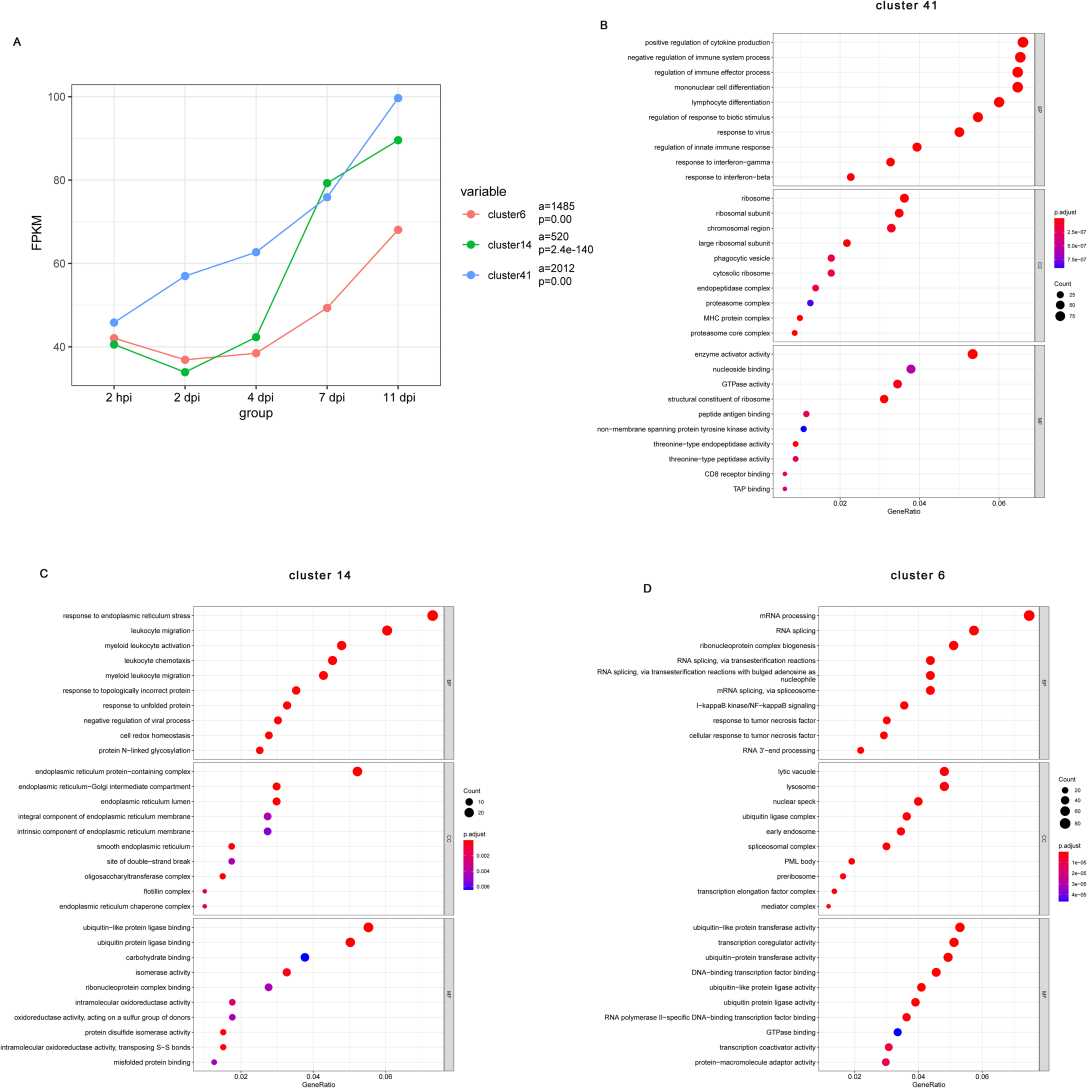
Short Time-series Expression Miner (STEM) analysis of the DE gene expression patterns during *B pertussis* infection. **(A)** Changes in all genes in Cluster 41, Cluster 14, and Cluster 6. The colored lines show the trends in gene expression changes over time after infection relative to the preinfection sample. The lowercase letter “a” indicates the quantity of genes in the corresponding cluster, and “p” indicates a statistically significant p value. **(B-D)** Gene Ontology (GO) enrichment analysis showing the top 10 biological process, cellular component, and molecular function terms enriched in the DE genes from Cluster 41 **(B)**, Cluster 14 **(C)**, and Cluster 6 **(D)** in the nasal turbinates of mice at each infection timepoint compared with preinfected mice (n = 5). The size of the dot indicates the number of genes with the corresponding GO annotation, and the color intensity indicates the adjusted p value of the corresponding GO term. The top 10 most significantly enriched GO terms related to the DE genes are shown.

Given that functional enrichment analyses of the transcriptional changes at each dpi relative to baseline does not consider the dynamic and longitudinal patterns of gene expression, we next used Short Time-series Expression Miner (STEM) to identify clusters of genes, the expression of which changes in a similar manner over time. The results of STEM showed that a total of 50 gene expression profiles were identified, including 13 statistically significant gene profiles (P< 0.05, highlighted with a colored background) (**Supplementary Figure S4**). Notably, the average expression of genes in Cluster 41 (2012 genes), Cluster 14 (520 genes), and Cluster 6 (1485 genes) increased significantly throughout the infection (**Figure 3A**).

To obtain a more detailed understanding of the functions of the genes in these clusters, we performed GO annotation analysis. According to the GO annotations, the top biological process terms enriched among genes in Cluster 41(2012 genes) were related to the innate and adaptive immune responses such as “regulation of cytokine production”, “mononuclear cell differentiation”, “lymphocyte differentiation”, “response to virus”, “response to interferon-gamma”, and “response interferon-beta” (**Figure 3B**). Genes also enriched to GO term “phagocytic vesicle”, “cytosolic ribosome”, “MHC protein complex”, “peptide antigen binding”, and “CD8 receptor binding” (**Figure 3B**). Cluster 14 (520 genes) was comprised of genes that enriched to GO terms indicative of innate immune regulation state such as “leukocyte migration”, “leukocyte chemotaxis”, and “myeloid leukocyte migration” (**Figure 3C**). Interestingly, for the consistently upregulated genes in Cluster 14, enriched cellular component terms and enriched molecular function terms mainly associated with the endoplasmic reticulum, including “endoplasmic reticulum protein-containing complex”, “endoplasmic reticulum-Golgi intermediate compartment”, “endoplasmic reticulum lumen”, and “endoplasmic reticulum lumen” (**Figure 3C**). Finally, upregulated genes of Cluster 6 (1485 genes) were mainly involved in innate inflammatory response such as “mRNA processing”, “I-kappa B kinase/NF-kappa B signaling”, “response to tumor necrosis factor”, “lysosome”, and “early endosome” (**Figure 3D**). These observations indicate changes associated with regulation of a series of genes that participate in the inflammatory response, immune response, and other processes.

### 3.4 High expression of type I IFNs production- and response-related genes throughout *B. pertussis* infection

**Figure 4:**
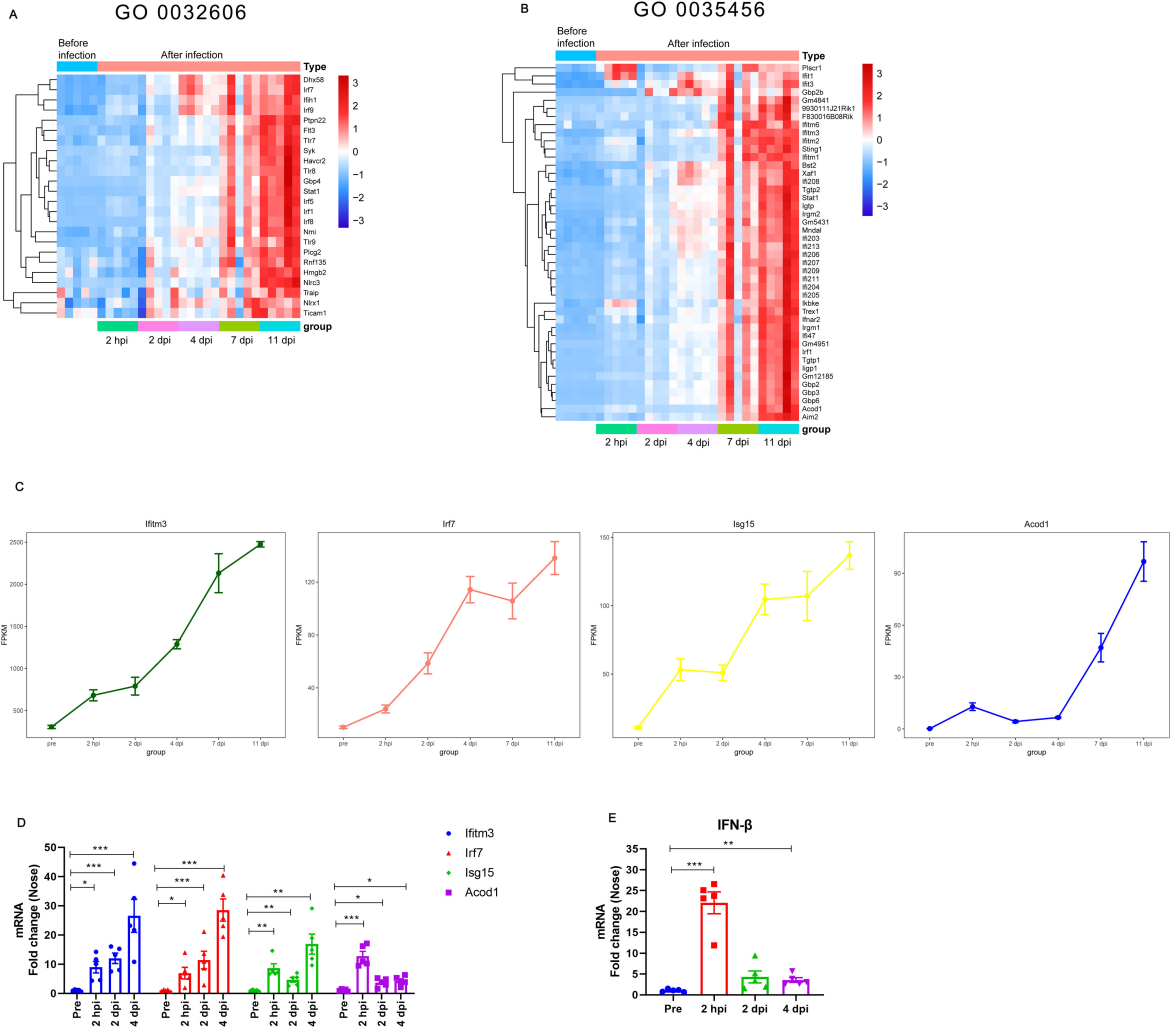
Expression of type I IFNs-related genes at different stages of *B. pertussis* infection in murine nasal turbinates. **(A-B)** Expression of type I IFNs-related genes obtained from RNA-seq at different stages of *B. pertussis* infection. Heatmap of genes enriched in the GO terms “type I IFNs production (GO 0032606)” **(A)** and “response to interferon-beta (GO 0035456)” **(B)**. Red indicates increased expression, and blue indicates decreased expression. **(C)** Average expression levels of the Ifitm3, Acod1, IRF7, and ISG15 genes obtained from RNA-seq at different stages during *B. pertussis* infection. **(D)** Validation of the Ifitm3, Acod1, IRF7, and ISG15 genes at 2 hpi, 2 dpi, and 4 dpi via RT−qPCR. **(E)** RT−PCR analysis of IFN-β gene expression levels in murine nasal mucosa at 2 hpi and 2 and 4 dpi. GAPDH was used as an internal reference gene. The asterisks indicate the level of significance (*, P< 0.05; **, P< 0.01; ***, P< 0.001; n = 5).

Given that we observed persistent upregulation of type I IFNs-related pathways in the analysis, we next analyzed type I IFNs-related gene transcript levels during *B. pertussis* infection. We focused on the DE genes enriched with the GO terms “type I IFNs production” (GO 0032606) and “response to type I IFNs” (GO 0035456). A heatmap was constructed to visualize the expression levels of type I IFNs-related genes in different stages (**Figures 4A**, **B**). As expected, *B. pertussis* infection was associated with increased expression of receptors associated with type I IFNs production, including *Tlr7*, *Tlr8, Tlr9*, *Dhx58, and Ifih1*(**Figure 4A**). Similarly, *B. pertussis* infection was also associated with increased expression of IFNs regulatory factors, *IRF1*, *IRF5*, *IRF7*, *IRF*8, and *IRF9* (**Figure 4A**). Additionally, the DE genes annotated with the enriched GO term “response to type I IFNs” included IFNs receptor (e.g., *IFNAR2*), immune signaling (e.g., *Acod1*, *Bst2*, *Aim2*, *Sting*, and *Irgm1*), IFNs-induced transmembrane (IFITM) proteins (e.g., *Ifitm1*, *Ifitm2*, *Ifitm3*, and *Ifitm6*), and IFNs-induced guanylate-binding proteins (e.g., *Gbp2b*, *Gbp3*, and *Gbp6*) (**Figure 4B**). Interestingly, *Ifitm3*, *IRF7*, *ISG15*, and *Acod1* (also known as immune-responsive gene 1, IRG1) were also among the 74 common genes that were differentially expressed at all 5 detection time points (**Figures 1B**, **4C**), indicating that type I IFNs signaling was high expression throughout *B. pertussis* infection. As expected, the average expression of the *Ifitm3*, *IRF7*, *ISG15*, and *Acod1* genes obtained from RNA-seq at different stages continuously increased (**Figure 4C**). Moreover, the expression levels of the four key genes were verified via RT−qPCR, and the relative expression levels were consistent with those in the RNA-seq data (**Figure 4D**). In addition, we selected several genes for validation, including *Stat1*, *Ifnar2*, *Aim2*, and *Sting* (**Supplementary Figure S5**). Lastly, we also detected IFN-β levels via RT−qPCR. As expected, we detected significant transcriptional upregulation of IFN-β after *B. pertussis* infection, which peaked at 2 hpi at approximately 22-fold greater than the level before challenge (**Figure 4E**). These results suggest that *B. pertussis* infection induces high expression of type I IFNs production- and response-related genes.

### 3.5 Type I IFN signaling has no effect on the bacterial burden in the respiratory tract but exacerbates the severity of *B. pertussis* infection

**Figure 5:**
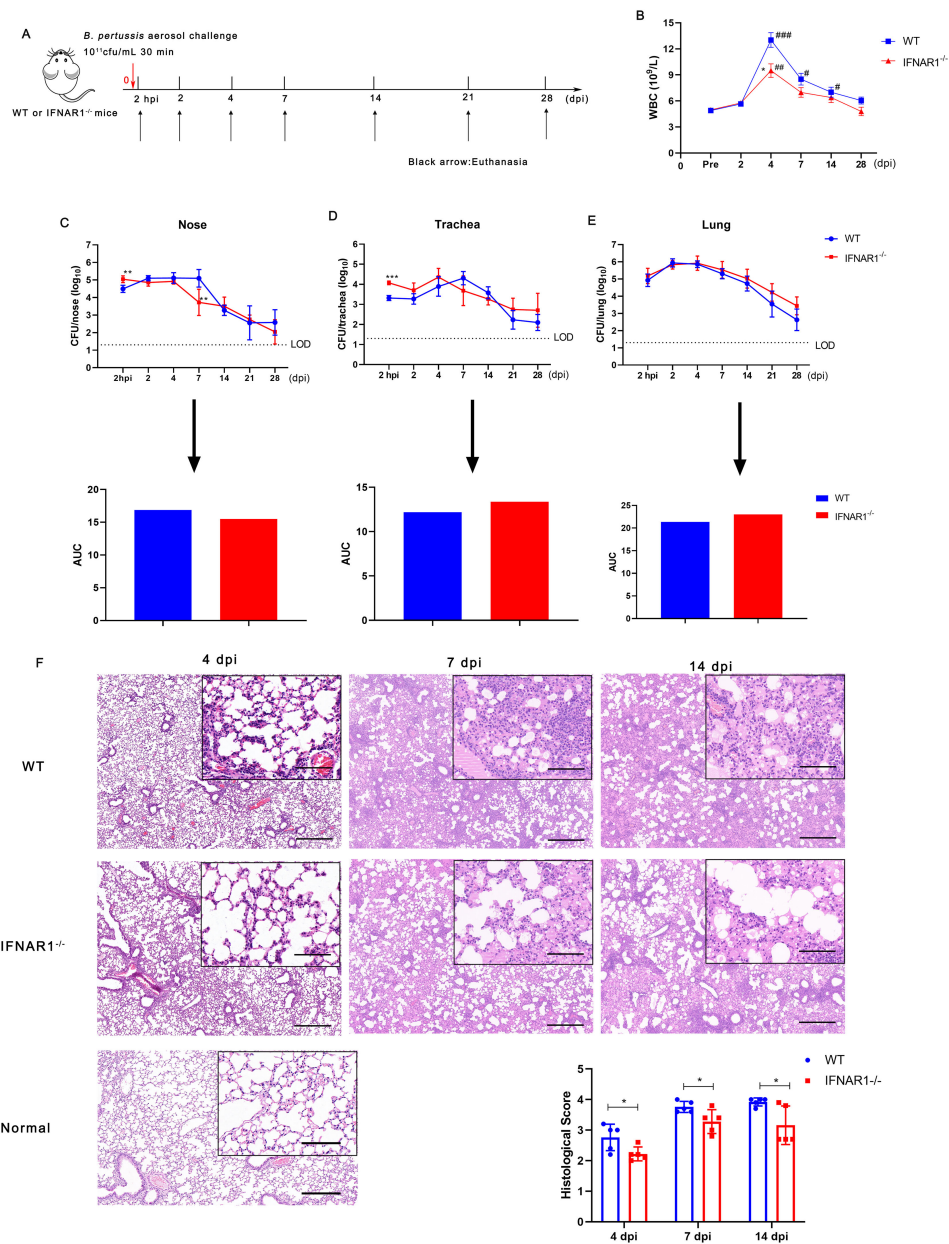
Leucocyte count, bacterial colonization, and lung histological changes in WT and IFNAR1^-/-^ mice after infection with *B. pertussis*. **(A)** Timeline of *B. pertussis* aerosol challenge and sample collection from WT and IFNAR1^-/-^ mice. **(B)** Dynamic profiles of the number of WBCs per μL of peripheral blood in *B. pertussis*-infected animals. **(C)** CFU counts in nasal homogenates and corresponding areas under the bacterial clearance curves (AUCs). **(D)** CFU counts in tracheal homogenates and corresponding AUCs. **(E)** CFU counts in the lung homogenates and corresponding AUCs. **(F)** Representative images of H&E-stained lung tissues from 4, 7, and 11 dpi. Scale bar, 50 μm. The results are presented as the means ± SEM (n = 5). The dashed line represents the lower limit of detection (LOD). The P value is indicated as follows: *P < 0.05, ** P < 0.01, *** P < 0.001 vs. WT mice; # P < 0.05, ## P < 0.01, ### P < 0.001 vs. prior to infection in the WT and IFNAR1^-/-^ groups.

*B. pertussis* infection induces an intense type I IFNs response; therefore, we further investigated whether type I IFNs influences the process of *B. pertussis* infection. Wild-type (WT) and IFNAR1 KO (IFNAR1^-/-^) mice were infected with *B. pertussis* via aerosol exposure (**Figure 5A**). We first assessed the changes in peripheral WBCs in mice after infection. At 4 dpi, the number of peripheral WBCs in both WT and IFNAR1^-/-^ mice was greater than that in preinfected mice; however, the total WBC count was significantly lower in IFNAR1^-/-^ mice than in wild-type mice (**Figure 5B**). Next, we compared the kinetics of *B. pertussis* clearance over the 2 hpi and 2, 4, 7, 14, 21, and 28 dpi timepoints between WT and IFNAR1^-/-^ mice. Nasal bacterial colonization in IFNAR1^-/-^ mice was greater than that in WT mice at 2 hpi but significantly lower than that in WT mice at 7 dpi, and no significant difference was detected at other time points compared with that in the WT group. The areas under the bacterial clearance curve (AUCs) in WT and IFNAR1^-/-^ mice were 16.87 and 15.49, respectively (**Figure 5C**).

With respect to tracheal infection, tracheal homogenates from WT mice were heavily colonized at 2 hpi after *B. pertussis* challenge and reached the highest level, 2.0 × 10^5^ CFU/mL, at 7 dpi; then, the number of colonies gradually decreased, with an AUC of 12.19 (**Figure 5D**). However, compared with WT mice, IFNAR1^-/-^ mice had higher CFU counts at 2 hpi and reached the highest level, 2.3 × 10^5^ CFU/mL, at 4 dpi, with an AUC of 13.35 (**Figure 5D**). We also observed lung colonization of *B. pertussis* in WT and IFNAR1^-/-^ mice. Both groups of mice presented similar bacterial colonization and clearance curves without clearance within 28 days, with AUCs of 21.35 and 23.03, respectively (**Figure 5E**).

We then examined the histological changes in the lungs of IFNAR1^-/-^ and WT mice at 4, 7, and 14 dpi. Large amounts of inflammatory cell infiltration, especially polymorphonuclear granulocytes and monocytes, lung interstitial thickening, and severe bronchial obstruction, were observed in the lungs of WT mice after challenge (**Figure 5F**). However, substantially fewer inflammatory cells and relatively slight bronchial obstruction were observed in the lungs of IFNAR1^-/-^ mice compared with those of WT mice. Histological examinations of lung sections revealed that pathological damage increased with time in both WT and IFNAR1^-/-^ mice. However, histological section scoring revealed that lung lesion burden and severity were both alleviated in the lungs of infected IFNAR1^-/-^ mice compared with those of WT mice (**Figure 5F**). In addition, we examined the albumin level in BALF of IFNAR1^-/-^ and WT mice at 4 dpi. which is another parameter reflecting the lung injury. Albumin levels were significantly reduced in the BALF of IFNAR1^-/-^mice when compared with WT mice (**Supplementary Figure S6**). Collectively, these findings demonstrate that type I IFN signaling exacerbates lung pathology during *B. pertussis* infection in mice.

### 3.6 Lack of IFNAR1 signaling contributes to reduced respiratory tract inflammatory responses in mice during *B. pertussis* infection

**Figure 6:**
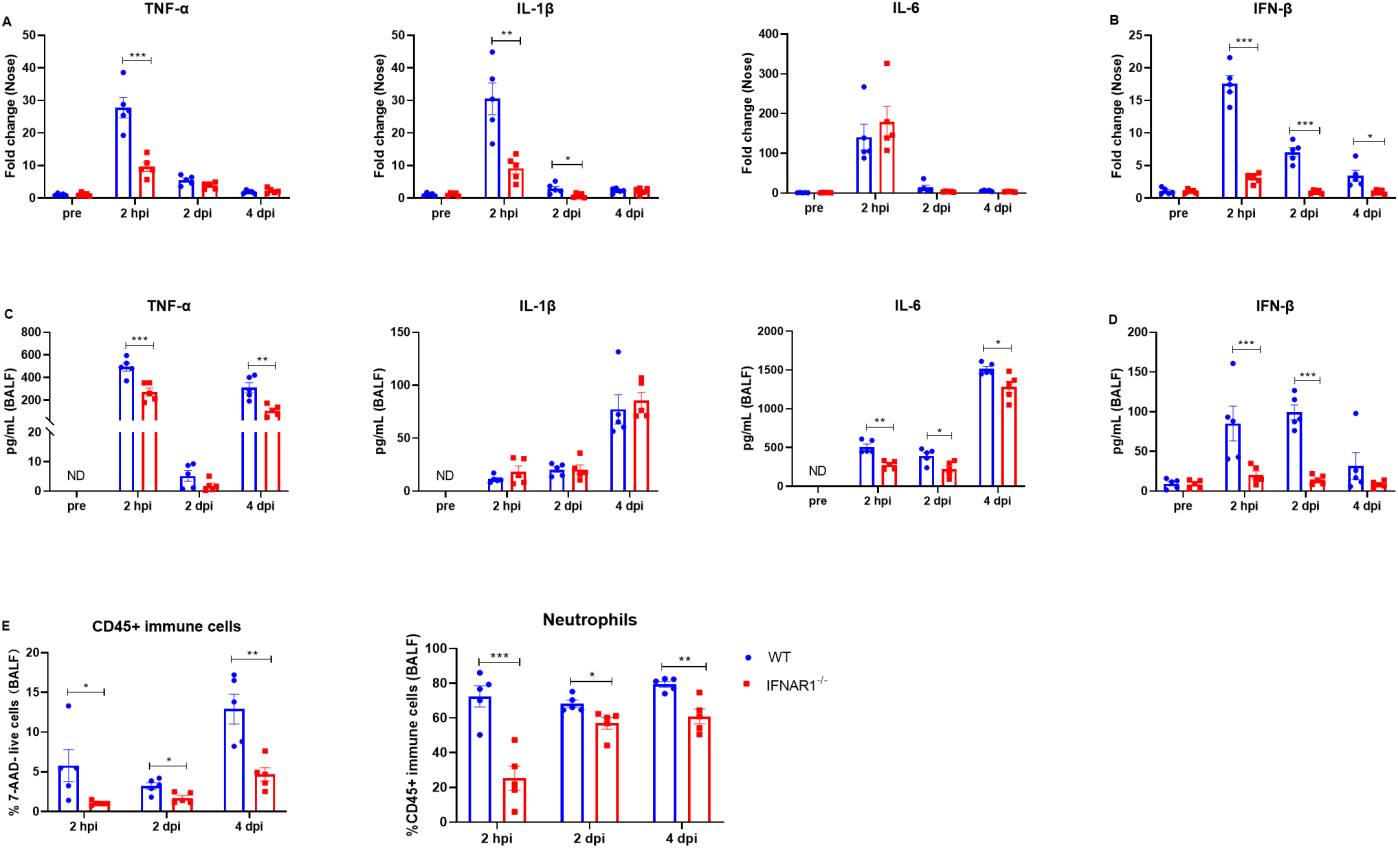
Inflammatory cytokine and neutrophil levels in WT and IFNAR1^-/-^ mice with *B. pertussis* infection. **(A, B)** Nasal homogenates from WT and IFNAR1^-/-^ mice infected with *B. pertussis* were analyzed for inflammatory cytokine (TNF-α, IL-1β, and IL-6) **(A)** and IFN-β **(B)** production at 2 hpi, 2 dpi, and 4 dpi (n = 5). **(C, D)** BALF from WT and IFNAR1^-/-^ mice infected with *B. pertussis* was analyzed for inflammatory cytokine (TNF-α, IL-1β, and IL-6) **(C)** and IFN-β **(D)** production at 2 hpi, 2 dpi, and 4 dpi (n = 5). **(E)** Proportion of CD45+ immune cells (7-ADD+CD45+) among all live cells and the proportion of neutrophils (CD45+CD11b+Ly6G+) among all leukocytes (CD45+ immune cells) in the BALF (for gating strategy, see **Supplementary Figure S7**). The data are expressed as the means ± SEM (n=5). * P < 0.05, ** P < 0.01, *** P < 0.001.

Next, we evaluated the effects of type I IFN signaling on the regulation of inflammatory cytokines. We measured the levels of multiple cytokines in the nasal turbinates and bronchoalveolar lavage fluid (BALF) at 2 hpi, 2 dpi, and 4 dpi via RT−qPCR and ELISA, respectively. We detected significantly higher mRNA levels of TNF-α in the turbinates of WT mice than in those of IFNAR1^-/-^ mice at 2 hpi (**Figure 6A**). In addition, IL-1β gene expression was significantly greater in WT mice than in IFNAR1^-/-^ mice at 2 hpi and 2 dpi (**Figure 6A**). IL-6 levels were comparable between WT mice and IFNAR1^-/-^ mice at the indicated timepoints (**Figure 6A**). We also measured the levels of IFN-β and observed that the level of IFN-β was significantly lower in the IFNAR1^-/-^ mice than in the WT mice (**Figure 6B**).

Next, we measured the protein levels of TNF-α, IL-1β, IL-6, and IFN-β in the BALF. IFN-β was present at very low levels in mice before infection (pre), while TNF-α, IL-1β, and IL-6 were undetectable (**Figures 6C, D**). There were no significant differences in the basal cytokine levels between WT mice and mice that lacked IFNAR1 (**Figures 6C, D**). Following *B. pertussis* infection, the level of TNF-α increased significantly in both WT mice and IFNAR1^-/-^ mice, but the level of TNF-α in the BALF was significantly lower in IFNAR1^-/-^ mice than in WT mice at 2 hpi and 4 dpi (**Figure 6C**). Similarly, the levels of IL-1β and IL-6 were also significantly increased after *B. pertussis* infection in both WT and IFNAR1^-/-^ mice. The levels of IL-1β in the two groups were comparable, whereas the level of IL-6 after infection was significantly lower in IFNAR1^-/-^ mice than in WT mice (**Figure 6C**). IFN-β was induced significantly (>50-fold) in WT mice after infection. IFN-β was induced at a very low level at 2 hpi and was sustained at a similar level on days 2 and 4 postinfection in infected IFNAR1^-/-^ mice, in contrast to the case in infected WT mice (**Figure 6D**). Furthermore, neutrophils in the BALF were assessed via flow cytometry at 2 hpi, 2 dpi, and 4 dpi. The percentage of CD45+ immune cells and neutrophils (gating strategy shown in **Supplementary Figure S7**) was significantly greater in WT mice than in IFNAR1^-/-^ mice (**Figure 6E**). Collectively, these results indicate that type I IFN signaling contributes to inflammatory reactions during *B. pertussis* infection.

### 3.7 Specific antibody levels and cytokine signatures suggest that a lack of IFNAR1 signaling does not influence the adaptive immune response during *B. pertussis* infection

**Figure 7:**
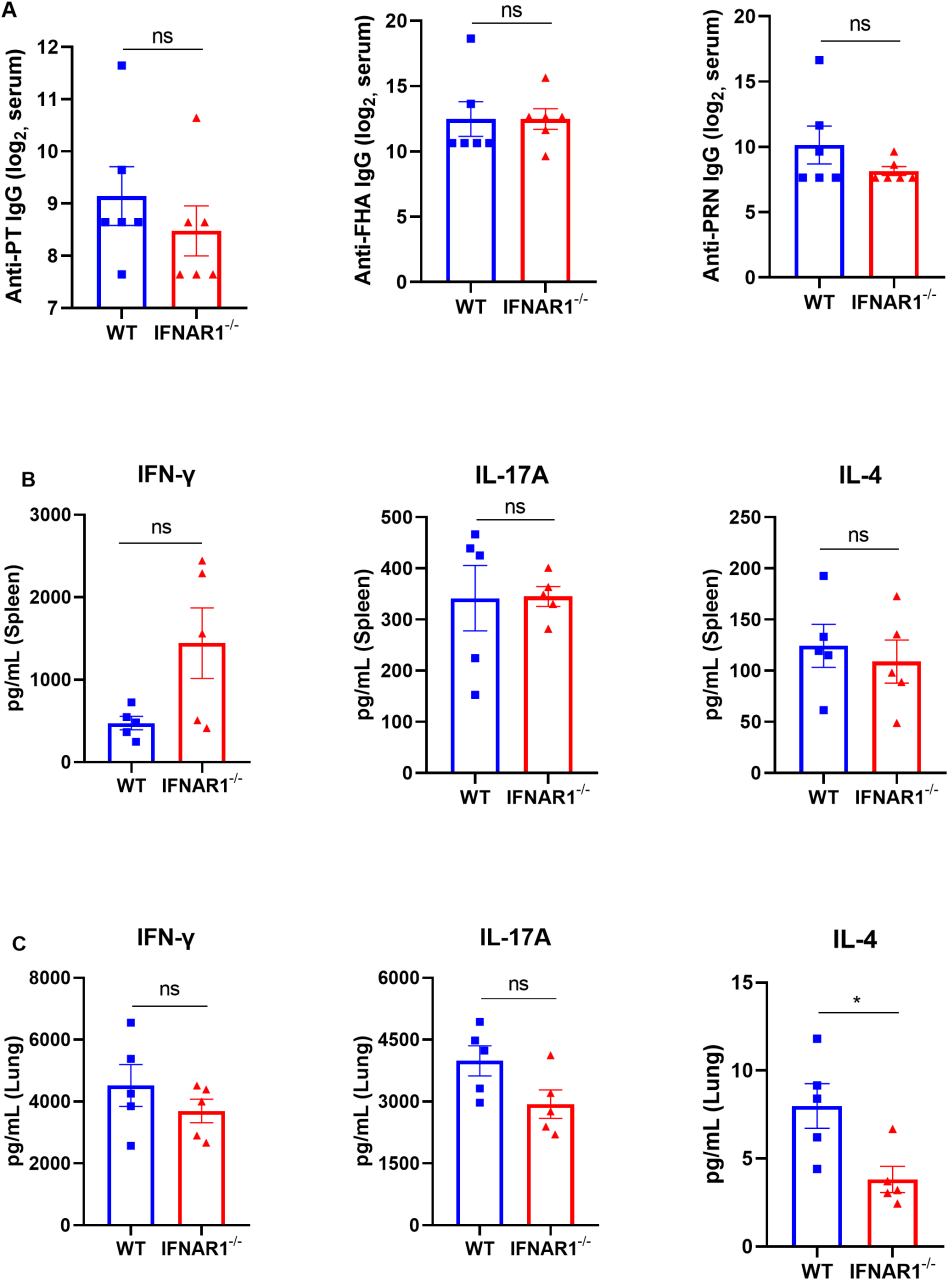
Serological responses to PT, FHA, and PRN and cytokines in the supernatants of lung and spleen lymphocyte cultures from *B. pertussis*-infected WT and IFNAR1^-/-^ mice. Specific antibodies against *B. pertussis* and cytokines in the supernatants of lung and spleen lymphocyte cultures from *B. pertussis*-infected WT and IFNAR1^-/-^ mice. **(A)** Total serum IgG titers against pertussis toxoid (PT), filamentous hemagglutinin (FHA), and pertactin (PRN) at 28 dpi. **(B, C)** Splenic and pulmonary lymphocytes were isolated at 28 dpi. The cells were cultured at a concentration of 2 × 10^6^/ml at 37°C with 5% CO_2_ and stimulated with the *B. pertussis*-specific antigens PT (2 μg/mL), FHA (2 μg/mL), and PRN (2 μg/mL). After incubation for 3 days, the culture supernatant was collected, and the levels of IFN-γ, IL-17A, and IL-4 were assayed via ELISA. Th1 response-associated cytokines (IFN-γ), Th17 response-associated cytokines (IL-17A), and **(C)** Th2 response-associated cytokines (IL-4) in the culture supernatants of splenic lymphocytes **(B)** and pulmonary lymphocytes **(C)**. The data are expressed as the means ± SEM. The P value is indicated as follows: * P < 0.05, ns, no significance (n=5).

To evaluate the effects of type I IFNs during *B. pertussis* infection on adaptive immune responses during *B. pertussis* infection, we examined pertussis toxoid (PT), filamentous hemagglutinin (FHA), and pertactin (PRN)-specific antibodies in the serum and cytokines in the supernatant of pulmonary and/or splenic lymphocytes after 3 days of incubation with PT, FHA, and PRN. The results revealed that at 28 dpi, IFNAR1^-/-^ mice presented levels of PT-, FHA-, and PRN-specific IgG comparable to those of the WT mice (**Figure 7A**). Moreover, the levels of cytokines associated with Th1 responses (IFN-γ), Th17 responses (IL-17A), and Th2 responses (IL-4) were evaluated in the splenocytes of IFNAR1^-/-^ mice and WT mice, and no differences were detected between the groups (**Figure 7B**). We also did not observe any significant differences in the levels of IFN-γ and IL-17A in the culture supernatants of pulmonary lymphocytes between the groups, but differences in Th2 (IL-4) responses were observed (**Figure 7C**). Overall, these results suggest that there is no significant difference in the antibody responses or Th1 and Th17 responses in either the spleen or lungs between IFNAR1^-/-^ and WT mice.

## 4 Discussion

Pertussis is a vaccine-preventable contagious respiratory disease; however, there has been a resurgence in cases in recent years, including in countries with widespread vaccination. A deep understanding of the pathogenesis of pertussis and the host factors that contribute to it is important. Here, we present the transcriptional landscape of the host upper respiratory tract response to *B. pertussis* infection, demonstrating that it is a dynamic process characterized by increased expression of a set of ancute inflammatory responses and innate immune processes, including the induction of a type I IFNs response. Next, we investigated the effects of type I IFNs in *B. pertussis* infection through studies of IFNAR1 KO mice. The results suggest that the lack of IFNAR signaling exacerbates pulmonary immunopathology and reduces the production of inflammatory cytokines and the recruitment of neutrophils to the lung during *B. pertussis* infection.

While numerous investigations have employed transcriptome analysis in lung and spleen tissues during *B. pertussis* infection, we focused on the nasal mucosa at which the host initially encounters the pathogen (30, 31). Transcriptome analysis of nasal turbinates revealed that the first line of defense of natural immunity in the upper respiratory tract response to *B. pertussis* infection is a highly dynamic and complex process. Acute phase responses and chemotaxis were observed at the early time points (2 hpi), followed by innate immune responses, phagocytosis, antigen processing and presentation, and finally genes involved in the adaptive immune responses at later time points (11 dpi). This observation agreed with Raeven et al. (30) ‘s conclusion that comprehensively reveals the dynamic change process of the lung immune response to *B. pertussis* infection, including the innate stage, transition stage, and adaptive stage. However, Deng et al. (32) found that there were temporal and spatial heterogeneities of the immune responses to *B. pertussis* infection in the lung and spleen of mice via analysis and modeling of dynamic microarray gene expression data. One of the main reasons why the nasal turbinates and lung tissues show certain similarities in the immune response to *B. pertussis* infection may be that both are directly exposed to antigens/bacteria. But whether the activation of the local nasal mucosal immune system can impact the lung immune environment during *B. pertussis* infection need further research. In addition, both Raeven et al. (30) ‘s study and the current study revealed that a rapid increase in the expression of genes related to the inflammatory signaling pathway early in infection, indicating the initiation of an acute inflammatory response. These inflammatory signaling pathways play important roles in inflammatory factor production as part of the innate immune system (33, 34). Moreover, GO analysis revealed increased enrichment of genes related to “leukocyte migration” and “neutrophil migration” at 2 hpi but not at 2 or 4 dpi. This finding corroborates the work of Raeven et al. (30), which showed gene expression of cytokines and acute phase proteins and AMPs was temporary suppressed at 2 days after *B. pertussis* infection. Moreover, Andreasen et al. (35) found that pathways for neutrophil recruitment are activated in naive mice within 6 h after *B. pertussis* infection, whereas neutrophil influx into the airway does not occur until 1–2 days later. Studies have also revealed that PT induces this phenomenon by inhibiting neutrophil chemotaxis (36). The differential expression analysis across timepoints also revealed great changes of DE genes in expression during the 2 hpi to 2 dpi transition. The functional analysis revealed enrichment of a greater number of immune signaling pathways at 7 dpi than at 4 dpi. Accordingly, we postulate that *B. pertussis* infection induced acute inflammatory responses in a short period, leading to immunosuppression within the line 2–4 days, ultimately followed by increases in the levels of transcripts associated with immune system processes, including those affecting T-cell activation and development. Indeed, *B. pertussis* can produce an array of virulence factors with immunomodulatory properties that play important roles in host−pathogen interactions (10, 37, 38). Our results further indicate temporary suppression of the nasal turbinates immune system by *B. pertussis*.

In the current study, the high expression of type I IFNs in the nasal turbinates and lung tissues of mice infected with *B. pertussis* was observed. Consistent with our findings, Ardanuy et al. (31) also observed upregulation of type I and III IFNs in adult mice in response to *B. pertussis* infection. Generally, bacterial induction of type I IFNs occurs mainly upon stimulation of Toll-like receptor (TLR) recognition of bacterial molecules and TLR-independent recognition of molecules delivered to the host cell cytosol which included the rupture of bacteria-containing phagosomes, bacterial cyclic dinucleotides, nucleic acids, and other factors (39–41). In the current study, transcriptome analyses indicated that Toll-like receptor (TLR) receptors (Tlr7, Tlr8, and Tlr9) associated with type I IFNs production were significantly upregulated after infection. It has been evident that TLR7/8 can recognize to single-stranded RNA (ssRNA), whereas TLR9 recognize DNA containing unmethylated CpG motifs. After recognizing ligands, they activate the intrinsic signaling pathways and induces type I IFNs to mediate innate immune responses (15, 42). The RIG-I-like receptors (or RLRs) encompass three homologous members, including the RNA helicases retinoic acid-inducible gene I (RIG-I; also known as DEAD box polypeptide 58, DDX58), the melanoma differentiation-associated gene 5 (MDA5; also known as interferon induced with helicase C domain 1, IFIH1), laboratory of genetics and physiology 2 (LGP2; also known as DExH box polypeptide 58, DHX58), which are cytosolic RNA sensors that recognize and bind directly to nonself RNA and induce a type I IFNs response to many RNA viruses (43). However, recent studies showed that in addition to viruses, RLRs are also able to sense bacterial nucleic acids and lead to the expression of type I IFNs. For example, Pagliuso et al. (44) found that RIG-I recognized a complex composed of *Listeria monocytogenes* RNAs and a small bacterial RNA-binding protein Zea during *Listeria monocytogene* infection, thereby enhancing the release of IFN-β. And Schmolke et al. (45) found that RNA of *Salmonella enterica serovar Typhimurium* was able to activate production of IFN-β in a RIG-I-dependent way in nonphagocytic cells. Similarly, we observed that RLRs, including IFIH1 (MDA5) and DHX58 (LGP2), were significantly upregulated in murine nasal turbinates after *B. pertussis* infection. Additionally, cyclic diguanosine monophosphate (c-di-GMP), which functions as an important second messenger to regulate bacterial numerous biological processes, also induces type I IFNs expression in independent of known cytosolic sensors or TLRs (46). For example, stimulator of IFNs genes (STING) acts as an immune sensor by inducing a type I IFNs response by directly binding to c-di-GMP; and helicase DEAD box polypeptide 41 (DDX41) (a c-di-GMP-detection pattern recognition receptor) also senses and directly binds to c-di-GMP, which encourages connections with STING, enhances STING’s affinity for c-di-GMP (47–49). In this study, we also detected that STING was expressed at higher levels in *B. pertussis*-infected mice than in preinfected mice. Actually, the study of Ardanuy et al. (50) highlights the importance of TLR9 and STING in the induction of type I IFNs and promotion of inflammatory pathology during *B. pertussis* infection. Nevertheless, the rest of receptors mentioned above may also be involved in the production of type I IFNs during *B. pertussis* infection. Future experiments that might be done to evaluate the relative importance of these different pathways.

The critical role of type I IFNs in establishing an antiviral state has been well documented (51). However, the role of type I IFNs in bacterial infection is complicated and can be either detrimental or protective to the host. In the present study, pulmonary histopathological lesions and the levels of proinflammatory cytokines and inflammatory cells in the lungs of *B. pertussis*-infected mice were greatly reduced in the absence of IFNAR signaling. This finding aligns with the work of Ardanuy et al. (31), they demonstrated that type I IFN signaling is associated with increased proinflammatory cytokine expression and exacerbated lung inflammatory pathology in adult mice. Some pathogens can exploit the type I IFNs response as part of their pathogenic strategy, but the molecular mechanisms involved remain unclear. Smith et al. (52) showed that lung plasmacytoid dendritic cell (pDC)-derived type I IFNs inhibits Th17 responses during early *B. pertussis* infection, thus contributing to early pathogenesis and the prolonged the course of pertussis disease. However, given the reduced recruitment of neutrophils to the lungs in IFNAR1^-/-^ mice, we speculated that type I IFNs may also promote neutrophil-mediated pulmonary histopathological lesions during *B. pertussis* infection. For example, Teixeira et al. (53) found that type I IFNs signaling promoted tuberculosis (TB) pathogenesis by inducing neutrophil-mediated lung inflammation and NETosis during *M. tuberculosis* infection. And Cabrera et al. (54) recently reported evidence for type I IFNs as a priming stimulus for neutrophil inflammasomes, which drive inflammation during severe COVID-19. Furthermore, Goritzka et al. (55) showed that type I IFNs signaling acts as a central driver of early proinflammatory responses in the lung during RSV infection. In this study, we found that the induction of proinflammatory cytokines (IL-1β, IL-6, and TNF-α) was abrogated in IFNAR1^-/-^ mice after *B. pertussis* infection. The more molecular mechanisms behind *B. pertussis* exploitation of type I IFNs signaling needs further investigation. In summary, the results of this study suggest that the treatment of pertussis could benefit from dampening inflammation by inhibiting the effects of type I IFNs.

In conclusion, our research reveals the transcriptome dynamics of the nasal turbinates in response to *B. pertussis* infection. *B. pertussis* infection resulted in significant differential gene expression, with notable upregulation of the type I IFNs signaling pathway, which can have both positive and negative effects during pathogen infection. Furthermore, we also reported that a lack of type I IFN signaling reduces the recruitment of neutrophils to the lung, alleviates pathology, and reduces the production of proinflammatory cytokines in the early acute phase of the disease but has no effect on adaptive humoral immunity or cellular immunity in the late phase of infection. Further investigations targeting specific molecules downstream of the type I IFNs pathway triggered through interactions between B. pertussis and the host defense system will open novel directions for therapy in addition to antibiotic treatment. However, the current study has several limitations. We acknowledge that mouse model may not fully capture the complexities of human disease, further studies need to focus on non-human primate or clinical data. Additionally, we focus on the transcriptome dynamics of the nasal turbinates and found a notable upregulation of the type I IFNs signaling pathway, but we did not reveal that whether the type I IFNs produced by the nasal turbinates are the most important factors that aggravate the inflammation of *B. pertussis* infection.

## Data Availability

The data presented in the study are deposited in the Sequence Read Archive (SRA) repository. The accession number is PRJNA1182990.
