# Supplementary material for: Bordetella pertussis infection activates the type I interferon signaling pathway to exacerbate respiratory tract inflammatory response
Source: Front Immunol. 2025 Mar 7;16:1521970. doi: 10.3389/fimmu.2025.1521970 (PMC11925776; doi:10.3389/fimmu.2025.1521970)
Supplement: Supplementary file 1 [file DataSheet1.docx]

Supplementary Material


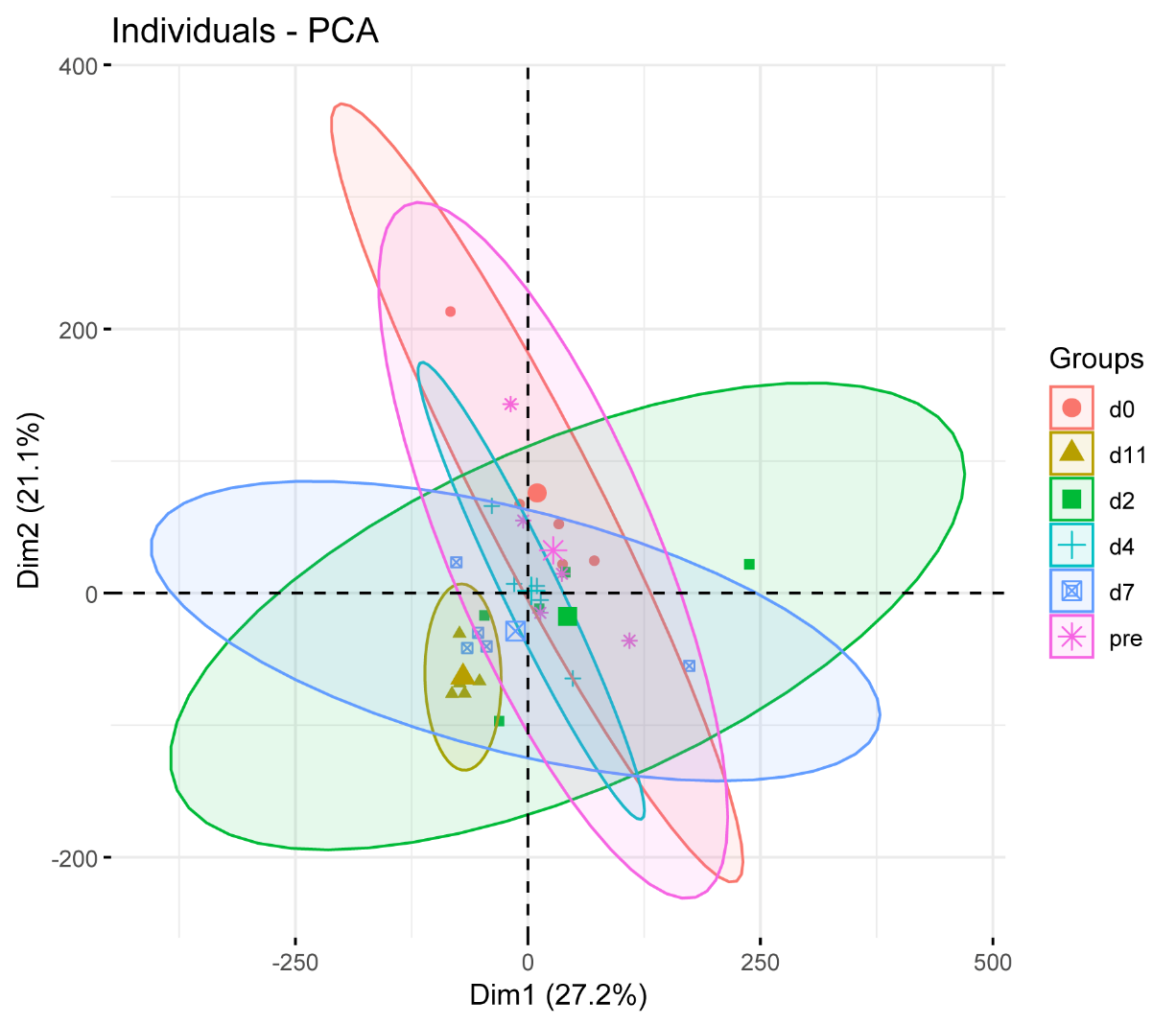


**Supplementary Figure 1. Principal component analysis (PCA) plot using the RNA-seq raw counts.**


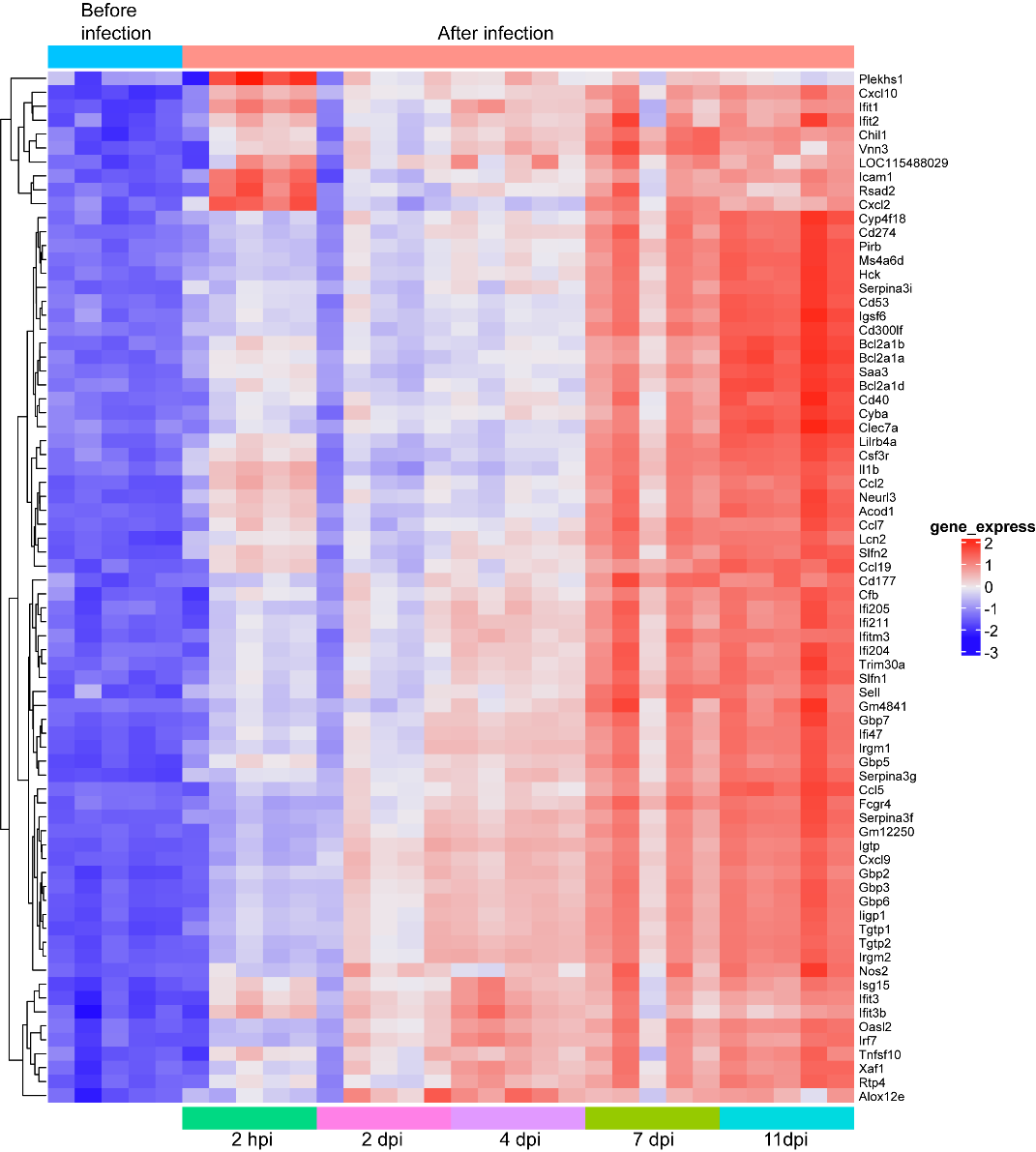


**Supplementary Figure 2. A heat map displaying the 74 DE genes consistently up- or downregulated at at 2 hpi, 2, 4, 7, and 11dpi.** The color value represents log2 (fold change).


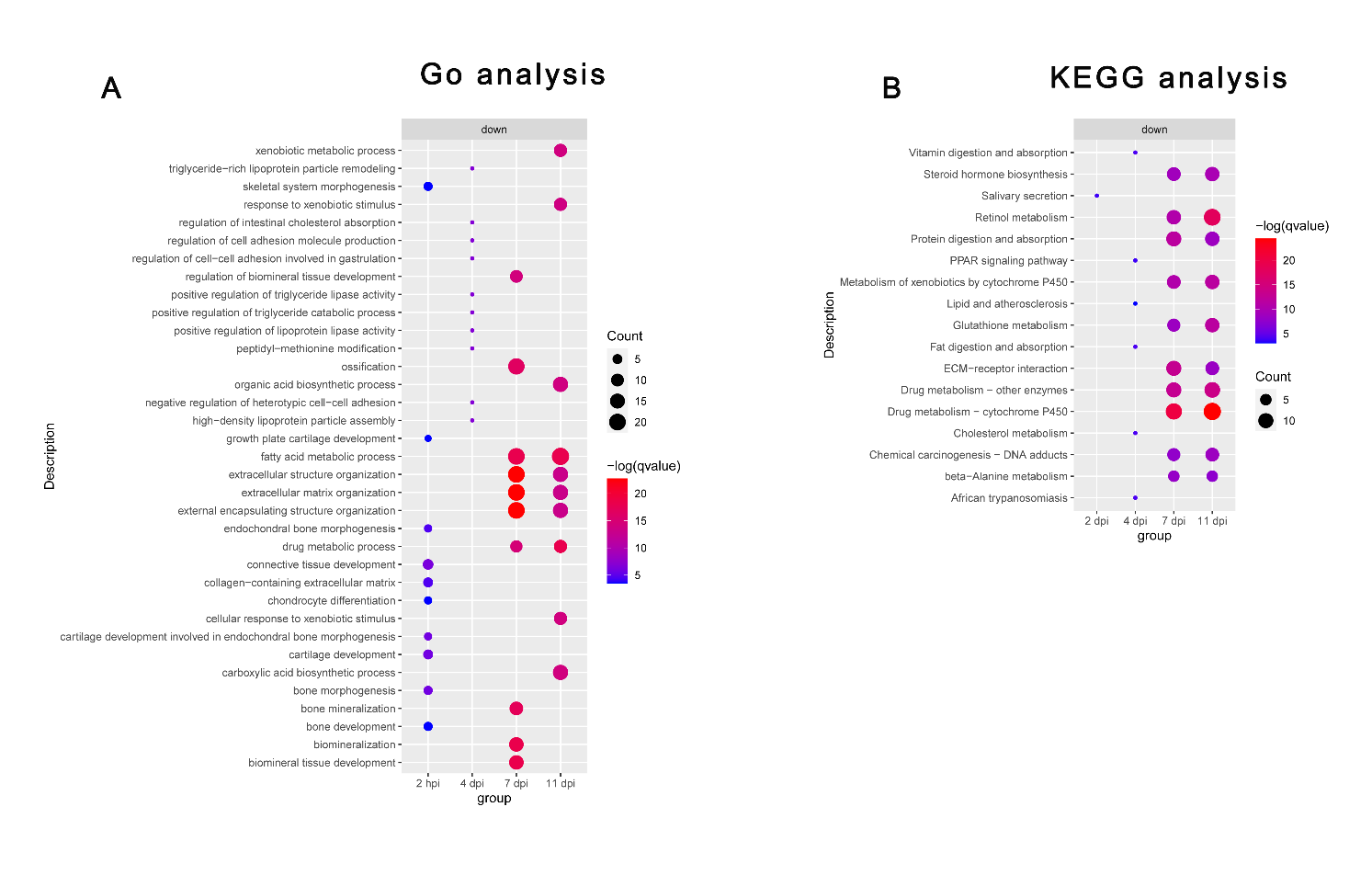
**Supplementary Figure 3.** **Functional Enrichment analysis of differential gene expression following *B. pertussis* infection in murine nasal turbinates.** Bubbleplot summarizing functional enrichment of DE Genes downregulated 2 hpi, 2, 4, 7, and 11dpi. Color intensity of each bubble represents the negative log of the FDR-adjusted p-value [–log(q-value)], and the relative size of each bubble represents the number of DE genes annotated with the specified Gene Ontology (GO) terms (A) and KEGG pathways (B). The top 10 most significantly enriched GO terms and KEGG pathways related to the DE genes for each time point are shown.


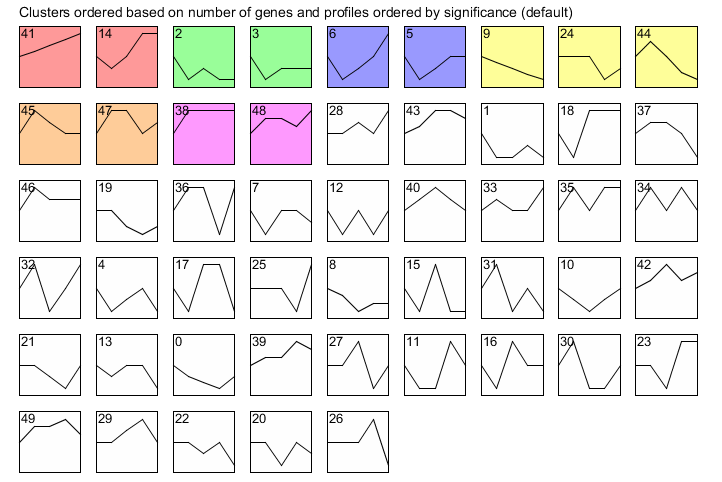


**Supplementary Figure 4. Short time series expression miner (STEM) analysis of DEGs at different stages.** Colorful squares represent significantly enriched profiles (P-value < 0.001), while Black-and-White ones represent nonsignificant trends. The black line represents the general tendency in each profile.


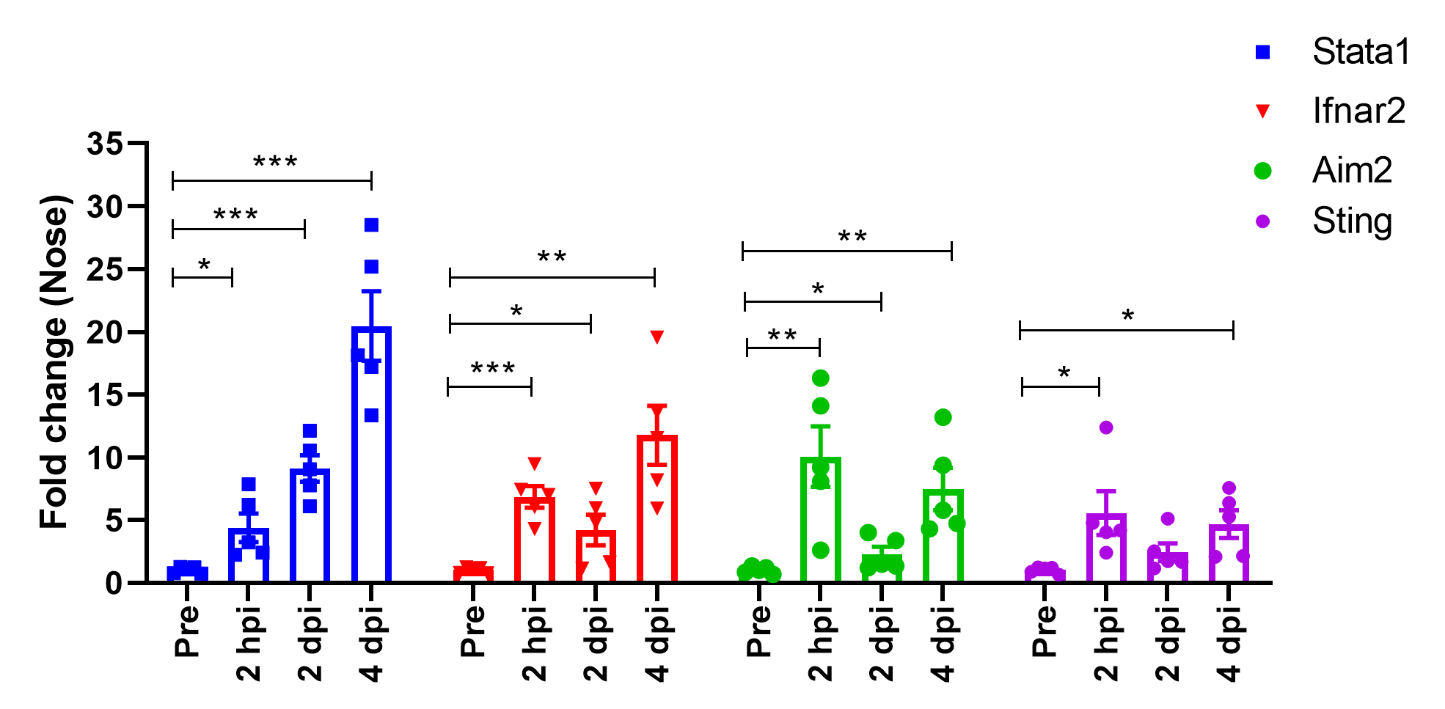


**Supplementary Figure 5. DE genes levels of type I IFNs-related genes.** Validation of Stata1, Ifnar2, Aim2, and Sting genes at prechallenge (pre), 2 hpi, 2 dpi, and 4 dpi via RT-qPCR. GAPDH was used as an internal reference gene. The asterisks indicate the level of significance (*, P< 0.05; **, P < 0.01; ***, P < 0.001; n = 5).

**
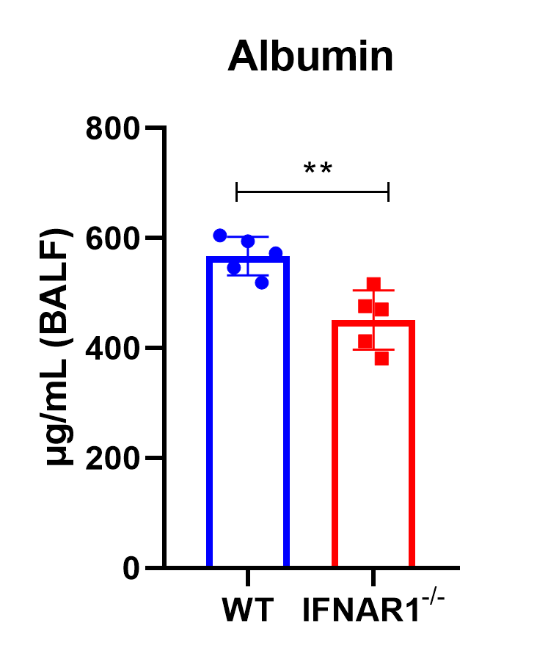
**

**Supplementary Figure 6. The concentration of albumin in BALF.** The concentration of albumin in the BALF was quantified by ELISA at 4 dpi and shown as mean ± SEM. The P value is indicated as follows: ** P < 0.01 (n=5).

**
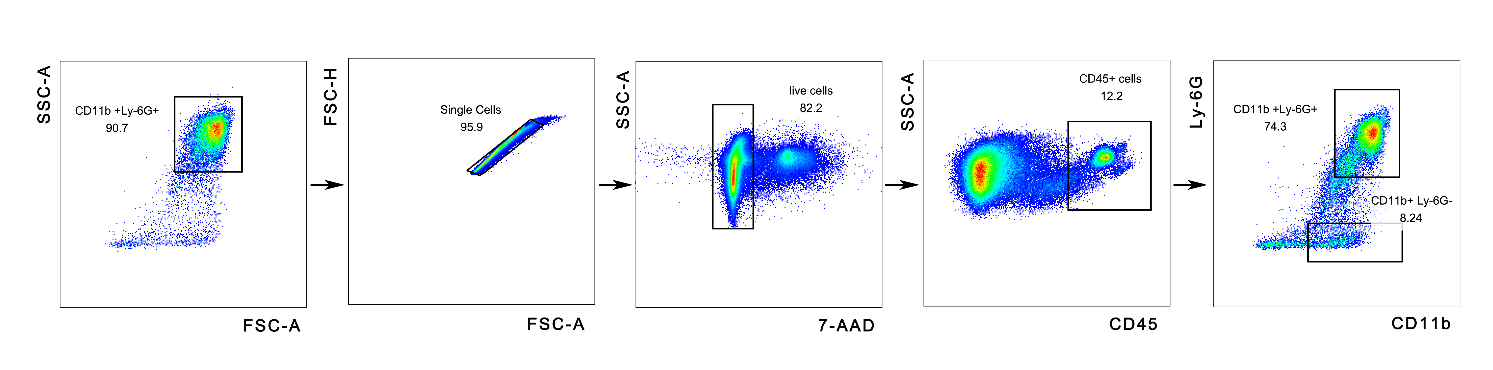
**

**Supplementary Figure 7. Gating strategy used to isolate** **CD45+CD11b+Ly6G+ neutrophils.** Live single lymphocytes were gated based on 7-AAD staining. Immune cells were identified based on CD45+ expression. Neutrophils were gated based on the marker expression patterns of CD45+CD11b+Ly6G+. These data are shown in Figure 6E.

**Supplemental Table 1. Primer sequences of each primer used**

| Primers | Sequence (5’-3’) |
| --- | --- |
| GAPDH forward | AGGTCGGTGTGAACGGATTTG |
| GAPDH reverse | GGGGTCGTTGATGGCAACA |
| Acod1 forward | GCGAACGCTGCCACTCA |
| Acod1 reverse | ATCCCAGGCTTGGAAGGTC |
| Sting forward | GGTCACCGCTCCAAATATGTAG |
| Sting reverse | CAGTAGTCCAAGTTCGTGCGA |
| Stat1 forward | TCACAGTGGTTCGAGCTTCAG |
| Stat1 reverse | CGAGACATCATAGGCAGCGTG |
| Ifnar2 forward | TACACCCTCTGGTACACAGTC |
| Ifnar2 reverse | CACTTATCTGTCACGTCACATGA |
| Irf7 forward | CCCCAGCCGGTGATCTTTC |
| Irf7 reverse | CACAGTGACGGTCCTCGAAG |
| Ifitm3 forward | CCCCCAAACTACGAAAGAATCA |
| Ifitm3 reverse | ACCATCTTCCGATCCCTAGAC |
| ISG 15 forward | GGTGTCCGTGACTAACTCCAT |
| ISG 15 reverse | TGGAAAGGGTAAGACCGTCCT |
| IFN-β Forward | TCCGAGCAGAGATCTTCAGGAA |
| IFN-β Reverse | TGCAACCACCACTCATTCTGAG |
| TNF-α forward | CCACGTCGTAGCAAACCACC |
| TNF-α reverse | CGGCTGGCACCACTAGTTG |
| IL-1β forward | TCTATACCTGTCCTGTGTAATG |
| IL-1β reverse | GCTTGTGCTCTGCTTGTG |
| IL-6 forward | CTGCAAGAGACTTCCATCCAG |
| IL-6 reverse | AGTGGTATAGACAGGTCTGTTGG |
